# Biofabrication and characterization of flavonoid-loaded Ag, Au, Au–Ag bimetallic nanoparticles using seed extract of the plant *Madhuca longifolia* for the enhancement in wound healing bio-efficacy

**DOI:** 10.1007/s40204-019-0110-0

**Published:** 2019-02-21

**Authors:** Mukti Sharma, Saurabh Yadav, Narayanan Ganesh, Man Mohan Srivastava, Shalini Srivastava

**Affiliations:** 1 Department of Chemistry, Faculty of Science, Dayalbagh Educational Institute, Agra, 282005 India; 2 Jawaharlal Nehru Cancer Hospital and Research Centre, Bhopal, 462001 India

**Keywords:** *Madhuca longifolia* seeds, Flavonoid loaded nano-biomaterials, Characterization of nano-biomaterials, Wound healing activity

## Abstract

**Abstract:**

The present communication warrants the presence of significant wound healing bio-efficacy of aq. alc. extract of the seed (49.78%) of the plant *Madhuca longifolia*. A family of seven flavonoid fractions have been ascertained in the seed aq. alc. extract of the target plant using LCMS-8030 analysis. In vivo wound healing parameters (wound area, wound closure, epithelization period, skin breaking strength and hydroxyproline content) have been examined in *Swiss albino* mice models. Statistically significant (*p* < 0.001) enhancement in the wound healing bio-efficacy has been effectively induced using flavonoid-loaded gold: (M_l_f@AuNp_s_)_,_ silver: (M_l_f@AgNp_s_), and Au–Ag bimetallic: (M_l_f@Au–AgNp_s_) nanoparticles. Among the biofabricated nano-biomaterials, M_l_f@AgNp_s_ exhibited an exceptional enhancement in the wound healing bio-efficacy (80.33%) attaining almost to the level of reference drug Placentrex (84.02%). All the fabricated nano-biomaterials were thoroughly characterized using UV–Vis, XRD, FE-SEM, TEM, EDX, and DLS. The promising enhancement in the wound healing potential of the nano-biomaterial (M_l_f@AgNp_s_) has been explained based on the cumulative effects of biological and nanotech parameters. The bio-fabricated (M_l_f@AgNp_s_) nano-biomaterials using the plant *M. longifolia* have lustrous prospects for the development of complimentary herbal nanomedicine for scaling-up the wound healing bio-efficacy.

**Graphical abstract:**

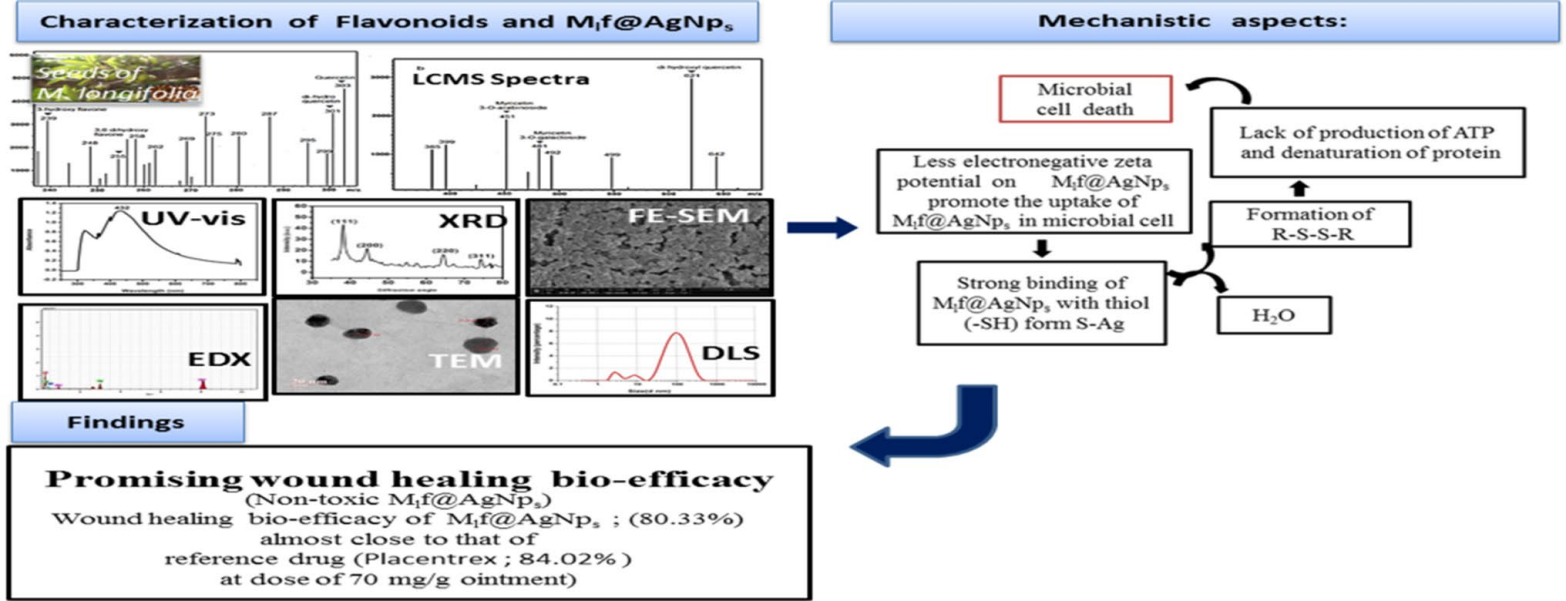

## Introduction

The wound is a rupture in the epithelial integrity of the skin-based structural changes and functions of tissues. Three important phases have been concomitant with the wound healing process (inflammation, cellular proliferation, and remodeling phase). Impaired wound healing results in severe morbidity leading to long hospitalization of patients. There is always demand for treating wounds for minimization of the time taken for healing and to step down the risks of undesired complications (Ahmadi and Adibhesami 2017; Kandhasamy et al. 2017). The use of conventional synthetic drugs over a long time is affiliated with side effects such as coma, hallucinations, kidney, heart, and liver failure (Biondi-Zoccai et al. 2006). The medicinal plants have been borne witnessed as the paramount source of various phytochemicals used for the biogenic synthesis. The use of plant-based nanomaterials has been accounted as a practical approach with improved physico-biochemical properties and functionality (Khoobchandani et al. 2013; Katti 2016). The biogenic nanoparticles have shown promising potential as wound healing agents. The green nanotechnology is an open inquisitive field of research for the enhancement of bio-efficacy and has been exploited in the development of nanodrugs (Murugan et al. 2015; Singh et al. 2018).

Numerous variety of metal nanomaterials are being acquired using gold, zinc, titanium, magnesium, silver, and copper (Sharma et al. 2007; Raliya and Tarafdar 2014; Bhakya et al. 2016; Chung et al. 2017). Among the noble metals, silver and gold have been a focus of interest for pharmacological bio-efficacies (Elia et al. 2014; Fatimah 2016). Silver, in particular, has potent antimicrobial activity including antifungal, anti-oxidant, anti-inflammatory, and wound healing (Kumar et al. 2016). Further, bimetallization can often surpass the enhancement of the catalytic properties of the original single metal, which may not be achieved by monometallic nanoparticles. The bimetallic nanoparticles are likely to exhibit not only additive combination of the properties of two individual metals, but also demonstrate the synergistic effects of the two metals.

Plant-mediated nanoparticles are non-toxic and ecofriendly than chemically synthesized nanoparticles (Ahmed et al. 2016). Considering the rapid blossoming of nanomedicine, particularly in prevention, diagnosis, and treatment of chronic wounds, this innovative technology will be soon on our doorstep.

Recent realization that the plants having particular bio-efficacy should be explored and enhanced for other bonafide activities, have motivated us to enhance anti-inflammatory bio-efficacy of the plant *Madhuca longifolia* using seed extract saponin-loaded Ag nanoparticles (Sharma et al. 2018)*.* In continuation of our work on this plant; exploring wound healing bio-efficacy in the seeds of the plant *M. longifolia*, the present communication reports a facile green synthesis of seed-extracted flavonoid-loaded Ag, Au, and Au–AgNp_s_ bimetallic nanoparticles, characterization and statistically significant enhancement in wound healing bio-efficacy. The observed highest enhancement in the wound healing bio-efficacy of M_l_f@AgNp_s_ has been ascribed to the inherent antimicrobial property of silver, nanosizing, biological factors responsible for higher uptake, and coating of medicinally important flavonoid on the nanoparticles.

*Madhuca longifolia* (Sapotaceae family) is grown in hot and damp climates of India. There is century’s old belief and observations of the medicinal uses of plant *M. longifolia* for skin-related issues (Mishra and Padhan^2013^; Sinha et al. 2017). In spite of its wide use over a long period of time, not much scientific approach has been made to study the wound healing activity of this plant at the nanoscale.

## Materials and methods

### Microwave–ultrasound assisted extraction

The plant seeds were collected from the village of Rajaborari, Madhya Pradesh, India and were identified by Taxonomy Division, Department of Botany, Dayalbagh Educational Institute, Agra, India, where the sample was deposited with the voucher specimen number DEI/DB/DH/2015-073. The defatted seed powder (250 g) was subjected to microwave-assisted extraction (200 W; 20 min; 25 °C) in aq. alc. solution and cooled. The extract was subjected to an ultrasonic bath for 40 min at room temperature, concentrated by rotavapor and dried with purging nitrogen.

### Isolation and characterization of flavonoids

The dried fraction of extract (25 g) was subjected to column chromatographic separation (length 120 cm; diameter 4 cm; stationary phase silica gel 125 g) and eluted with CH_3_Cl/CH_3_OH/H_2_O (70:30:1 v/v). After the removal of solvent, a brown mass was acquired. The brown mass fraction was subjected to LCMS-8030 for characterization of the flavonoid compounds. The experimental conditions were as follows: column; C18 column (4.6 mm × 150 mm, 2.5 µm), stationary phase; silica gel, mobile phase; 0.1% formic acid and 90.9% methanol, N_2_ nebulizing gas flow rate; 2 L/min, temp; 40 °C, injection volume; 0.2 µL scanning range (*m*/*z*); 100–1000; wavelength 254 nm followed by 15 min run time. The mass spectrometric analysis was performed in positive ESI mode.

### Biofabrication and characterization of bio-nanomaterials

Optimized experimental conditions of biofabricated nanoparticles were as follows: *Madhuca longifolia* flavonoid-loaded gold nanoparticles (M_l_f@AuNp_s_): At pH 5.5, 1 mL of flavonoid fraction (70 mg/mL) was mixed with 5 mL of hydrogen tetrachloroaurate dihydrate solution (HAuCl_4_·2H_2_O: 1mM) in a beaker and reaction mixture was subjected to sonication for 20 min at 20 kHz. *Madhuca longifolia* flavonoid-loaded silver nanoparticles (M_l_f@AgNp_s_): At pH 11.5, 1 mL of flavonoid fraction (70 mg/mL) was added with 10 mL of silver nitrate solution (1 mM) in a beaker and reaction mixture was subjected to sonication for 40 min at 20 kHz. *Madhuca longifolia* flavonoid-loaded bimetallic nanoparticles (M_l_f@Au–AgNp_s_): At pH 10, 1 mL of flavonoid fraction (70 mg/mL) was added to 10 mL of hydrogen tetrachloroaurate dihydrate solution (HAuCl_4_∙2H_2_O:1 mM), followed by the addition of 10 mL of silver nitrate solution (1mM) in a beaker and mixture was subjected to sonication for 40 min at 20 kHz. The formation of M_l_f@AuNp_s_, M_l_f@AgNp_s,_ and M_l_f@Au–AgNp_s_ were perceived by the change in color from pale yellow to ruby red, brown and pink, respectively.

The biofabricated nanoparticles were characterized using Ultraviolet/visible spectroscopy (UV–Vis 3000^+^ Lab India, India), X-ray diffraction (Bruker AXS D8 Advance, Germany), Field emission scanning electron microscopy (Nova Nano FE-SEM 450, Netherlands), Transmission electron microscopy, Energy dispersive X-ray spectroscopy (Tecnai G2 T 20 ST, Germany), and Dynamic light scattering (Nano ZS90 model Malvern, Germany).

### Formulation prior to topical application

Hard paraffin (25 g) and cetostearyl alcohol (25 g) were mixed and heated gently to 60 °C with constant stirring in a water bath to acquire a gel. White soft paraffin (425 g) and wool fat (25 g) were mixed together and allowed to cool. The optimized doses of the selected amount of reference drug, seed extract, flavonoid fraction, and biofabricated nanoparticles were added into per gram of this ointment and gently mixed.

### In vivo bioassay (excision and incision wound model)

Male *Swiss albino* mice (weight 25–30 g) were obtained from animal house of Jawaharlal Nehru Cancer and Research Centre Bhopal, Madhya Pradesh and used for the evaluation of in vivo experiments (vide Ethical permission; CPCSEA Registration no. 500/01/9/CPCSEA/2017)*.* The animals were kept at a temperature of 25–28 °C in clean polypropylene cages with 12 h light and dark cycles with proper pellet diet and water ad libitum. The mice were divided into seven groups, having six animals in each group. Group I served as control. Group II was treated with the reference drug (Placentrex; 70 mg/g ointment). The groups III and IV were treated with seed extract and flavonoid fraction at an optimized dose of 70 mg/g ointment. The groups V, VI, and VII were treated with M_l_f@AuNp_s_, M_l_f@AgNp_s,_and M_l_f@Au–AgNp_s_ at an optimized dose of 70 mg/g ointment. The posterior dorsal side hairs of the mice of all the groups were shaved. Animals were anesthetized prior to the creation of wound using the subcutaneous injection of local xylocaine (0.2 mL; 2% w/v). All the treatment groups along with reference drug, seed extract, flavonoids, and biofabricated nano-biomaterials were applied gently to cover the wounded area daily, until complete healing was achieved. In excision model, an area of 100 mm^2^ was carefully excised. The percentage of wound closure was calculated from the wound area (Mekonnen et al. 2013). The healing tissues were isolated on the 12th day from all the groups of the mice evaluated for histological investigation. The period of epithelization was calculated (Gutierrez and Vargas 2006) in terms of the number of days required for falling off the dead tissue remnants without any residual raw wound. In incision model, a longitudinal para vertebral incision of 3 cm in length was made deep through the skin. The wounds were closed with interrupted sutures 1 cm apart. The sutures were removed on the 8th day of post-incision and the treatment was continued. The skin breaking strength of the wound was measured on the 10th day after treatment (Kokane et al. 2009) with a tensiometer (Model: XU22DTF, Shanghai Lun Jie Mechanical and Electrical Co. Ltd., China 2000).

### Estimation of hydroxyproline content

On the 15th day, a piece of skin from the healed wound area of all the treatment groups was collected and analyzed for hydroxyproline content (Woessner 1961). The tissues (10 mg) were dried in a hot air oven at 60–70°C and hydrolyzed in 6 N HCl (5 mL) at 130 °C for 4 h in a sealed tube. The hydrolyzate was neutralized to pH 7.0, and was subjected to chloramine T oxidation for 20 min, and the reaction was terminated by the addition of 0.4 M perchloric acid (10 mL). The color developed by the addition of Ehrlich reagent (10 mL) at 60 °C was measured at 557 nm using UV–Vis spectrophotometer.

### Statistical analysis

The results were expressed as the mean ± SD of six animals. The data were analyzed by one-way ANOVA followed by Tukey test. The data were considered significant at *p* < 0.001. Statistical analysis was done using Graph Pad Insat 3.0 software.

## Results

### Presence of flavonoids

**Fig. 1:**
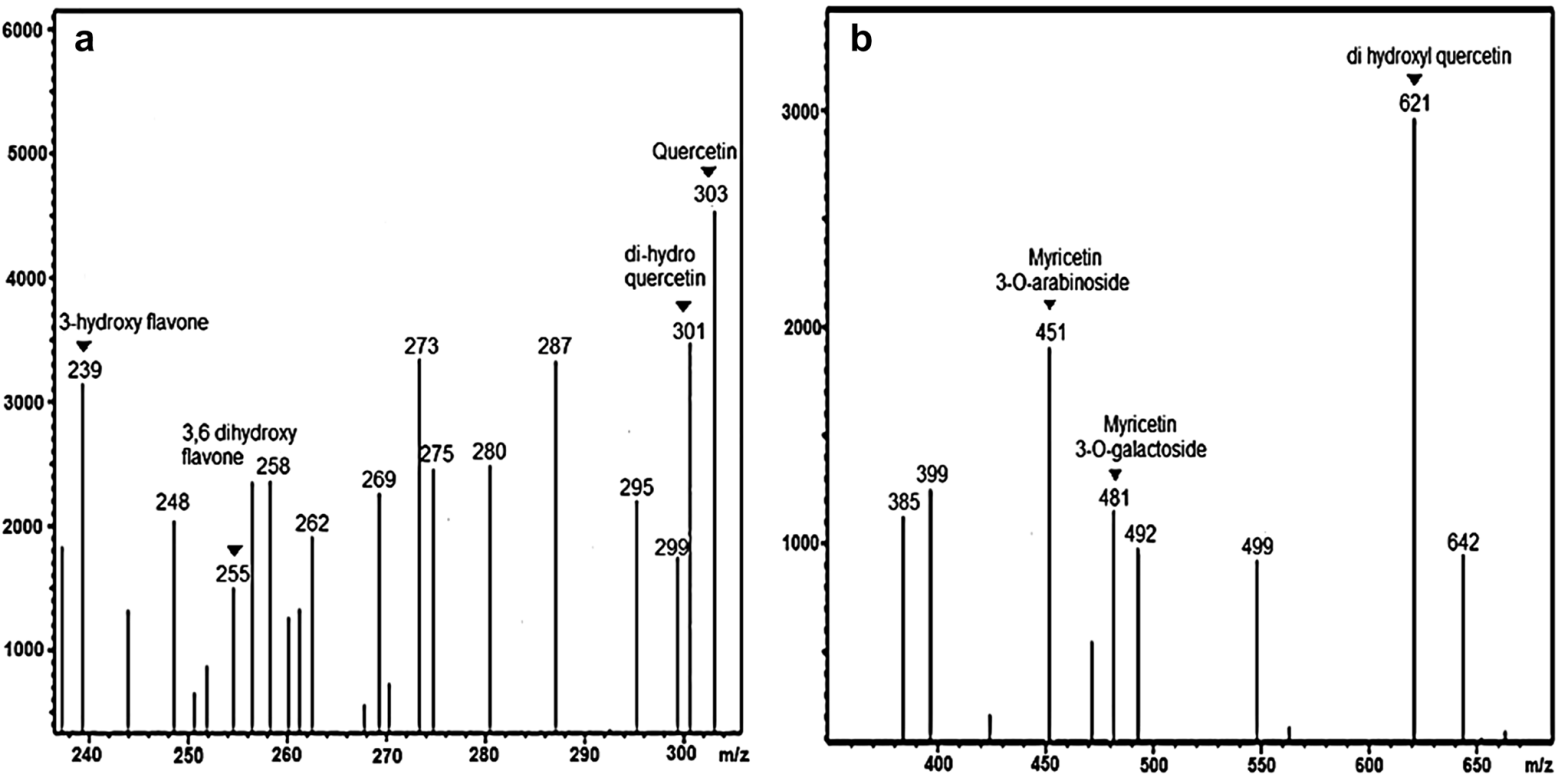
LCMS chromatogram of seven flavonoids in seed extract of the plant *M. longifolia* in the range **a** 230–310, **b** 350–700

LCMS-8030 chromatogram of the column chromatographic fraction of native seed extract scanned in the lower (230–310) and higher (350–700) ranges of *m*/*z*, exhibited the presence of 3-hydroxy flavones (*m*/*z*: 239), 3,6 dihydroxyflavone (*m*/*z*: 255), dihydroquercetin (*m*/*z*: 301), Quercetin (*m*/*z*: 303), Myricetin 3-O-arabinoside (*m*/*z*: 451), Myricetin 3-O-galactoside (*m*/*z*: 481) and dihydroxyl quercetin (*m*/*z*: 621) on the basis of their [M−H]^+^ mode (Fig. 1a, b).

### Characterization of biofabricated nano-biomaterials

#### UV–Vis spectroscopy

**Fig. 2:**
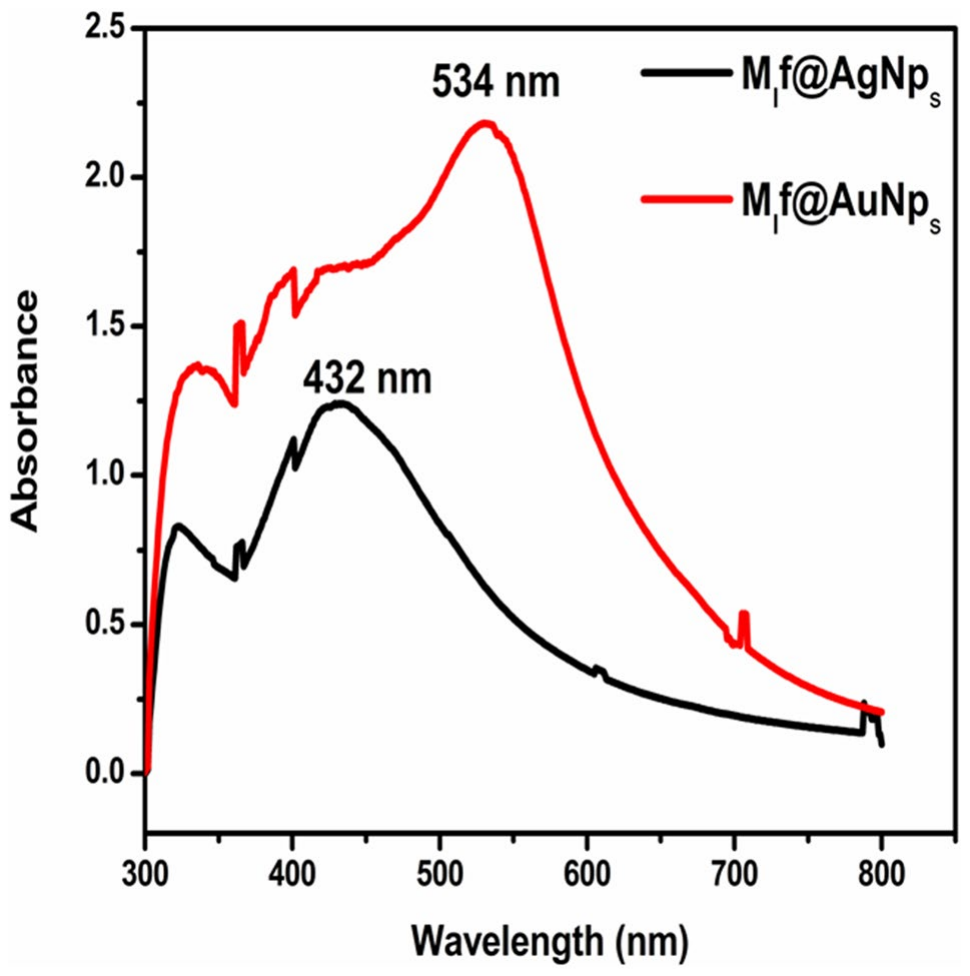
UV–Vis spectra of M_l_f@AuNp_s_ and M_l_f@AgNp_s_

The synthesis of M_l_f@AuNp_s_ and M_l_f@AgNp_s_was carried out at different concentrations (10^−4^–10^−2 ^M) of hydrogen tetrachloroaurate dihydrate and silver nitrate solution keeping the concentration of flavonoid constant (1 mL; 70 mg/mL) as a function of pH (2.5–13.5) in each case. The surface plasmon resonance bands at the concentration (10^−3^) of HAuCl_4_.2H_2_O at *λ*_max_ = 534 nm (pH 5.5) and AgNO_3_ solution at *λ*_max_ = 432 nm (pH 11.5) were considered optimum for the biofabrication of M_l_f@AuNp_s_ and M_l_f@AgNp_s_ because of their higher intensity (Fig. 2). The SPR bands at desirable *λ*_max_ may be ascribed to the coherent oscillation of the electrons in the conduction band of respective gold and silver nanoparticles.

**Fig. 3:**
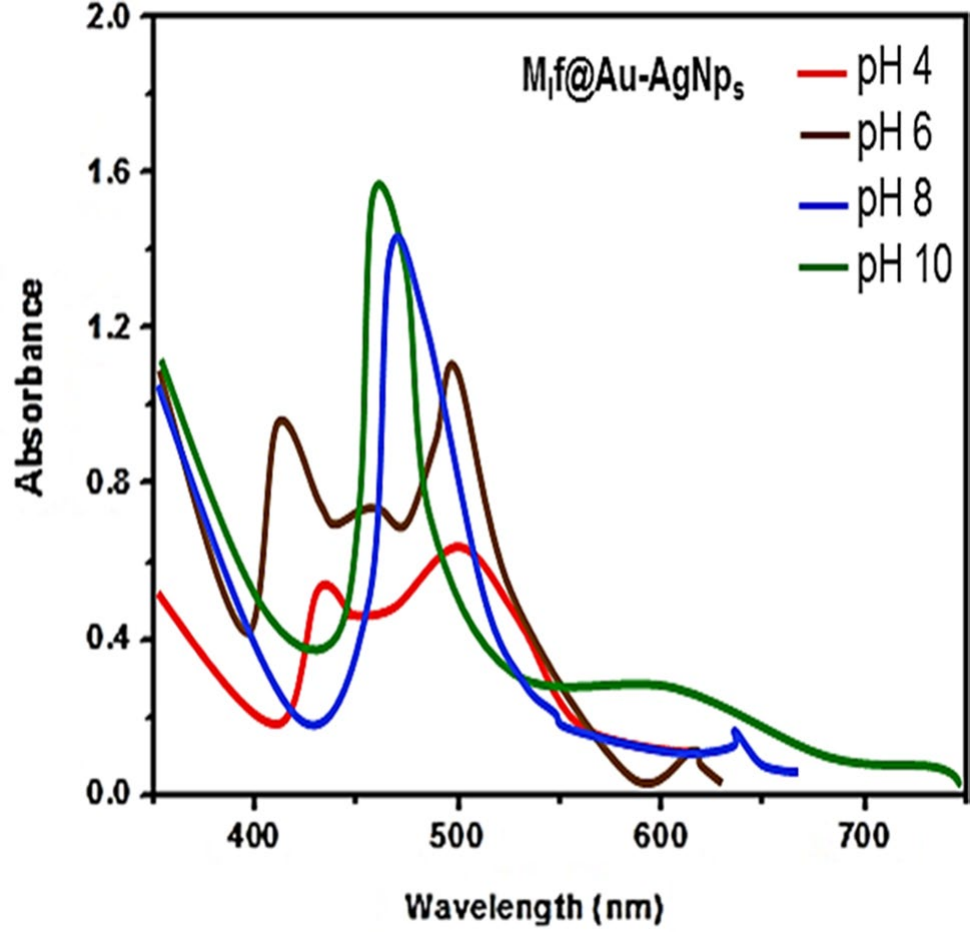
UV–Vis spectra of M_l_f@Au–AgNp_s_ bimetallic nanoparticles

Formation of Au–Ag bimetallic nanoparticles involved simultaneous co-reduction of Au(III) and Ag(I) solution. At pH 4 and 6, initially, a single peak of Au was obtained at characteristic wavelength 510 and 500 nm, respectively. At both the pH values with the passage of time, one more peak of Ag appeared at characteristic wavelength 430 and 420 nm. The delay in the newly generated peak may be attributed to the relatively slow formation of AgNp_s,_ highlighting the assembling of AgNp_s_ onto the surface of the AuNp_s_. At pH 8 and 10, a single peak at 470 and 460 nm appeared. However, no change was discerned at further higher pH. The hypsochromic shift from 470 to 460 nm at pH 10 attributed the formation of small-sized and more stable bimetallic nanoparticles (Fig. 3). The observation is in harmony with earlier observation (Ganaie et al. 2016), depicting a single peak of the formation of bimetallic Au–Ag nanoparticles at pH 10. The appearance of wide and shoulder bands in the UV–Vis spectra may be ascertained to the loading of flavonoids on the surface of biofabricated gold, silver, and bimetallic nanoparticles (Shaik et al. 2018). The fact has been supported on the basis of our TEM and DLS studies.

#### X-ray diffraction

**Fig. 4:**
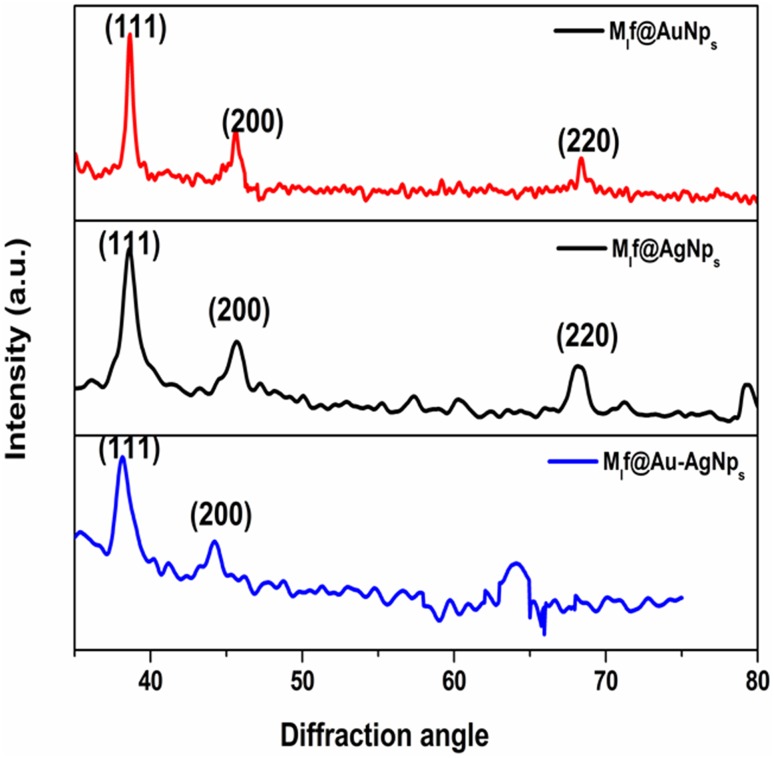
XRD of M_l_f@AuNp_s_, M_l_f@AgNps, and M_l_f@Au–AgNp_s_ bimetallic nanoparticles

The X-ray diffraction (XRD) profiles of all the biofabricated nanoparticles are depicted in Fig. 4. The three distinct peaks of biofabricated gold and silver nanoparticles were found at 38.18°, 44.39° and 64.57; 38.11° 44.27° and 64.42° diffraction angle, respectively. These peaks correspond to (111) (200) and (220) lattice planes of face-centered cubic structure of gold and silver nanoparticles (JCPDS file 04-0784, 04-0783). The bimetallic Au–AgNp_s_ had two diffraction peaks at diffraction angles 38.12° and 44.15°. It could be indexed to (111) and (200) having lattice planes of face-centered cubic structure of bimetallic Au–AgNp_s_. The intensity of the diffraction peak corresponding to (200) crystallographic plane was lower than (111). The 111 plane is known to be more reactive because of its high atom density (Cruz et al. 2010). Some unassigned peaks were also observed due to the crystallization of bio-organic phase (Niraimathi et al. 2013).

#### FE-SEM and EDX studies

**Fig. 5:**
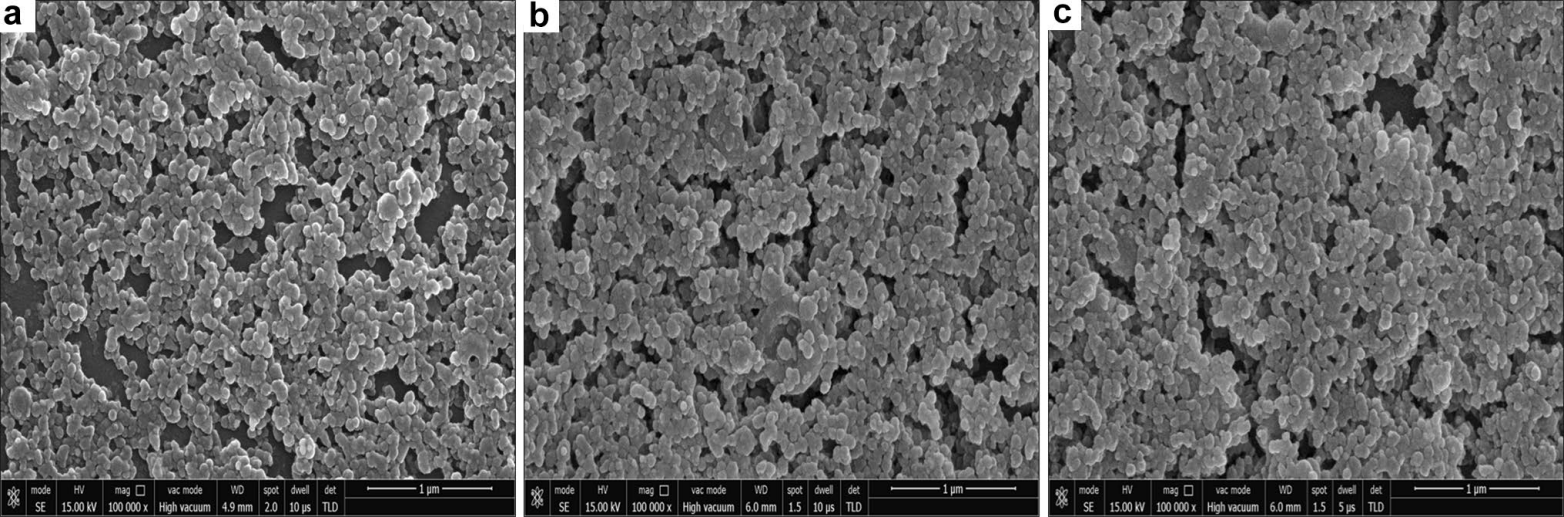
FE-SEM of M_l_f@AuNps, M_l_f@AgNp_s_ and M_l_f@Au–AgNp_s_ bimetallic nanoparticles

**Fig. 6:**
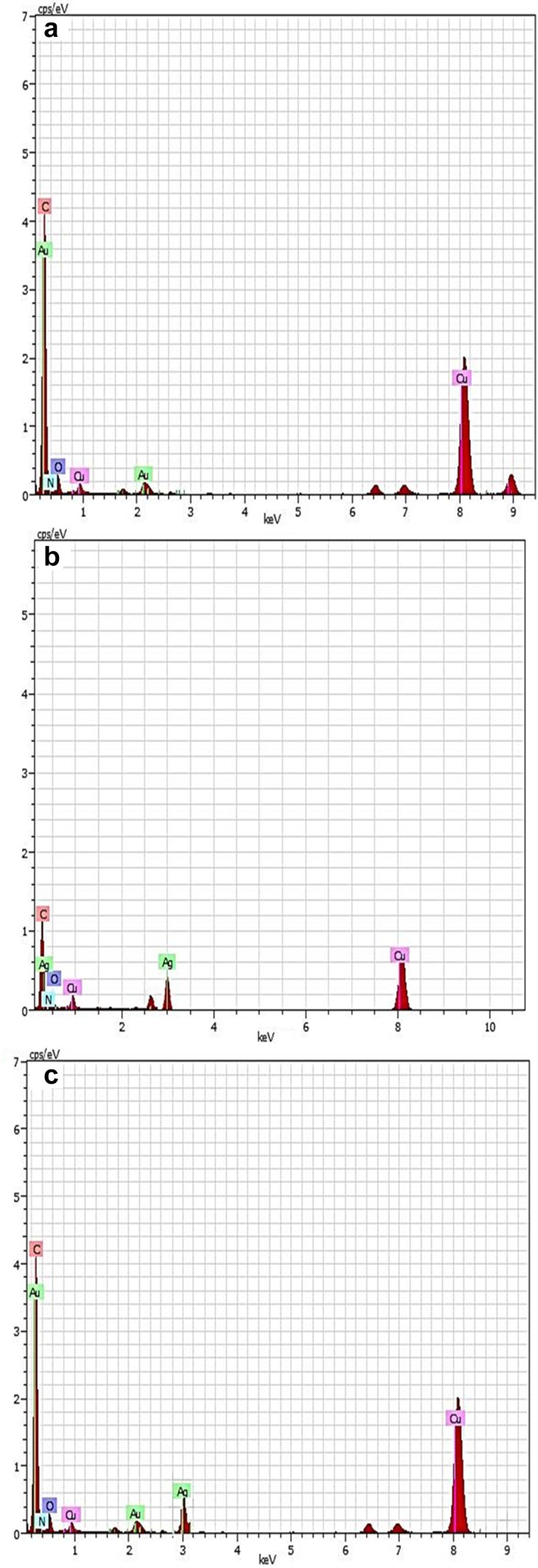
EDX of M_l_f@AuNp_s_, M_l_f@AgNp_s_ and M_l_f@Au–AgNp_s_ bimetallic nanoparticles

FE-SEM images (Fig. 5a–c) were acquired from drop-coated films of nanoparticles, indicated polydispersed spherical-shaped surface morphology of all the three biofabricated nanoparticles. The desirable signals of gold and silver metals were found in EDX spectra at 2 and 3 keV, respectively (Fig. 6a, b). Both characteristic peaks of Au and Ag at 2 and 3 keV were observed in the EDX spectra of Au–Ag bimetallic nanoparticles (Fig. 6c). The appeared peak of Cu was presumably related with Cu grid on which sample was coated. The peaks of C, O, and N might have initiated from the biomolecules that are adhered to the surface nanoparticles.

## TEM and DLS studies

**Fig. 7:**
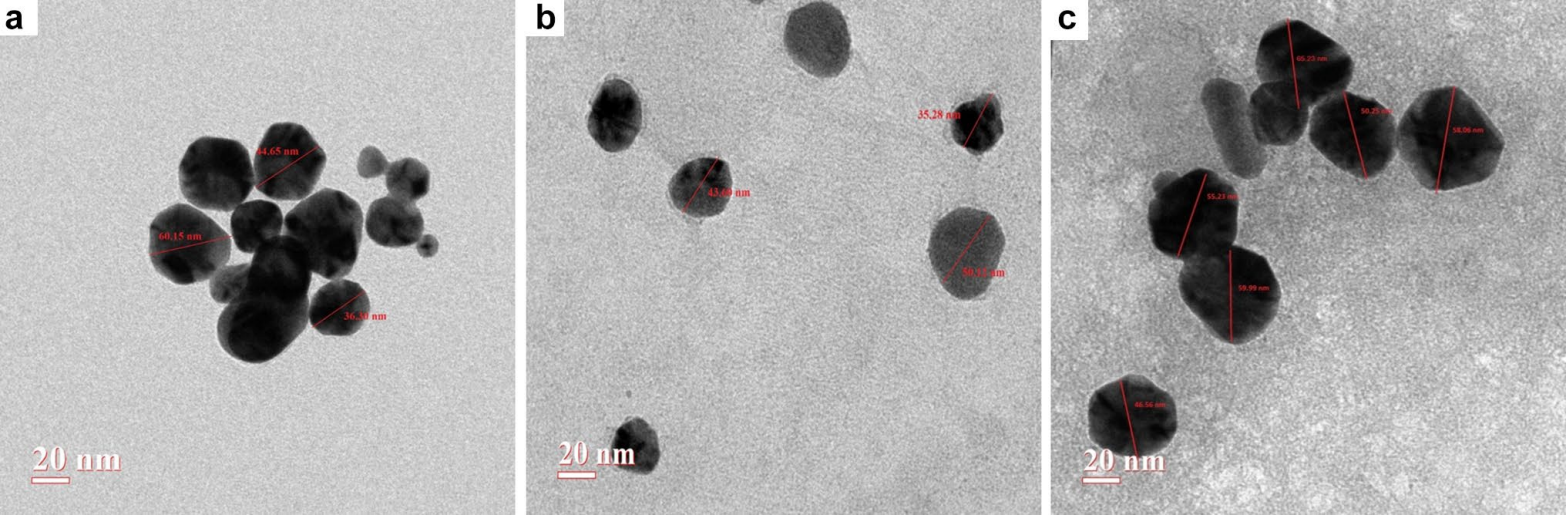
TEM of M_l_f@AuNp_s,_ M_l_f@AgNp_s_and M_l_f@Au–AgNp_s_ bimetallic nanoparticles

**Fig. 8:**
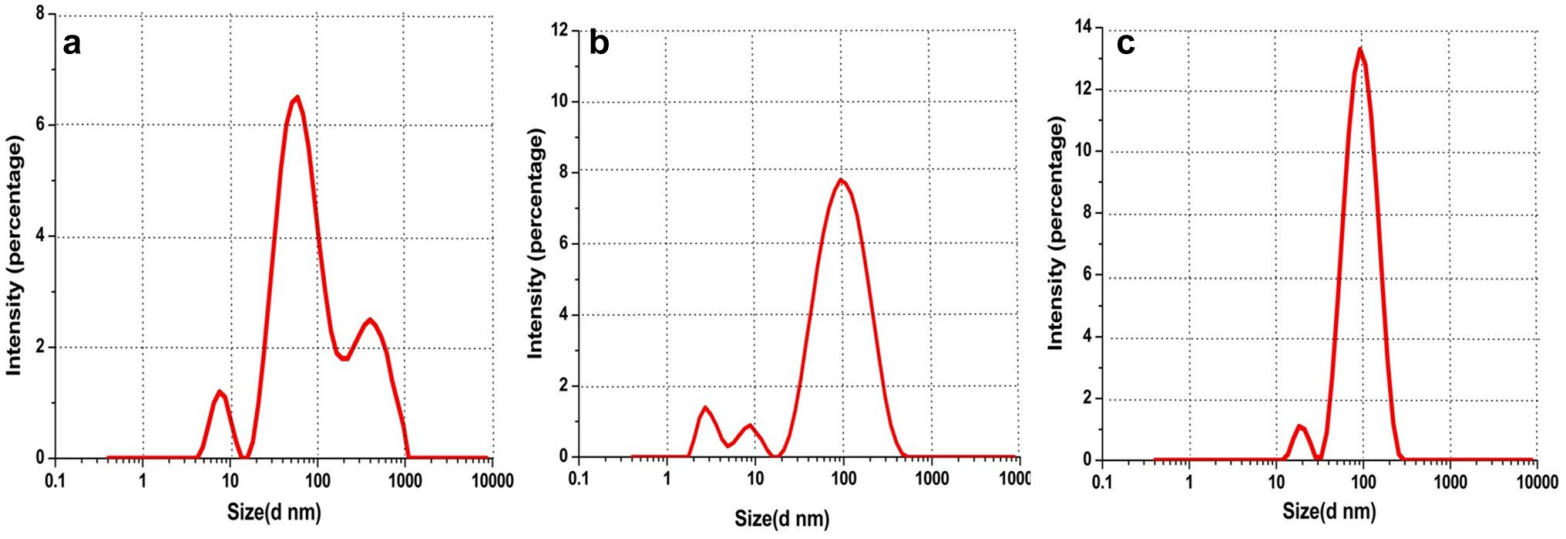
DLS size distribution curve of M_l_f@AuNp_s,_ M_l_f@AgNp_s_ and M_l_f@Au–AgNp_s_ bimetallic nanoparticles

**Fig. 9:**
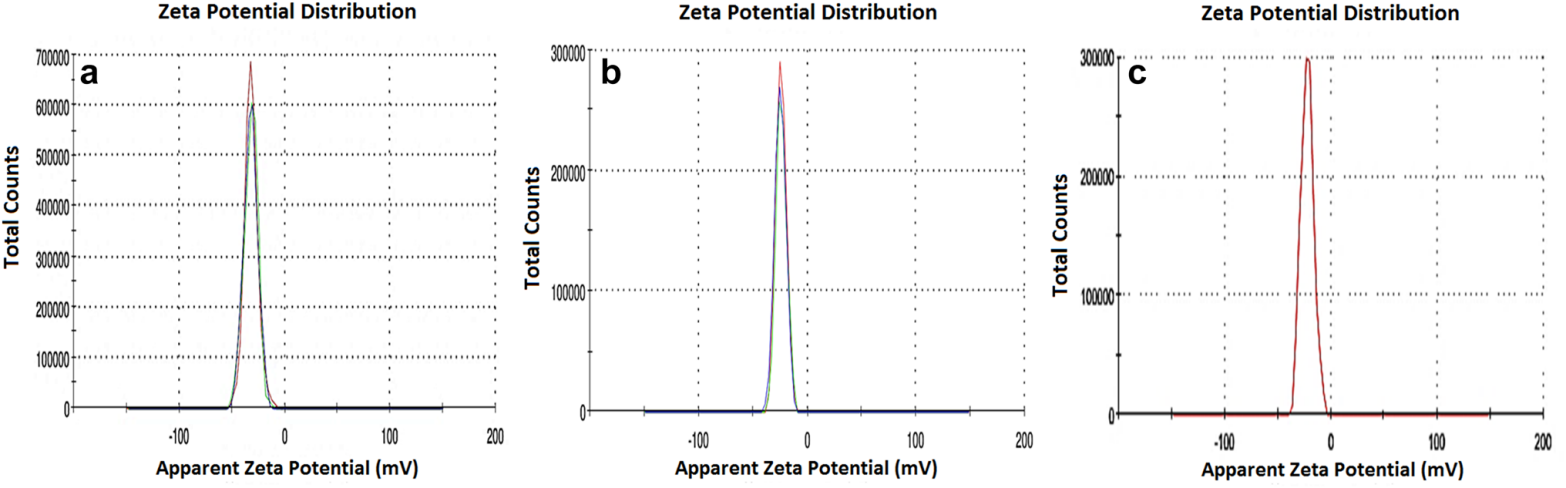
Zeta potential of M_l_f@AuNps, M_l_f@AgNp_s_ and M_l_f@Au–AgNp_s_ bimetallic nanoparticles

TEM analysis (Fig. 7a–c) also confirmed the spherical shape of all the three biofabricated nanoparticles having diameter range 36–60, 35–50 and 34–66 nm, at the magnification of 300,000×. The appearance of the faint thin layer around the nanoparticles in TEM images was the indication of the coating of secondary metabolites (flavonoids). The average hydrodynamic size (*Z* average given by DLS) of biofabricated nanoparticles, viz M_l_f@AuNp_s_, M_l_f@AgNp_s,_ and M_l_f@Au–AgNp_s_ were 74.20, 54.50 and 81.50 nm, respectively. An asymmetric distribution of these nanoparticles was as follows: 50–180, 13–170 and 15–185 nm (Fig. 8a–c). Size of the particles, appeared larger when measured by DLS as compared to the TEM. TEM provides the accurate size of nanoparticles but DLS delivers important information regarding the size distribution of particles. The difference possibly reflects the fact that TEM only measures the physical size while DLS measures the hydrodynamic size of the particles along with the ions attached to the surface and move with nanoparticles in solution (Huang et al. 2007; Cumberland and Lead 2009). The biofabricated nanoparticles M_l_f@AuNp_s_, M_l_f@AgNp_s_, and M_l_f@Au–AgNp_s_ exhibited zeta potentials as− 33.9, −22.5 and − 31.9 mV, respectively (Fig. 9a–c) are quite stable.

### In vivo bioassay (excision and incision wound model)

In vivo wound healing bioassay on *Swiss albino* mice was carried out with native seed extract in the various ranges (30, 50, 70 and 80 mg/g ointment). Based on the maximum wound healing potential (wound area, epithelization period, skin breaking strength, and hydroxyproline content in tissues), the dose of native seed extract was optimized (70 mg/g ointment). All the groups except control, dose (70 mg/g ointment) of reference drug, seed extract, flavonoid fraction, M_l_f@AuNp_s_, M_l_f@AgNp_s_, and M_l_f@Au–AgNp_s_ were provided. Table 1 includes various wound healing parameters in the excision and incision wound model at an optimized dose.

**Fig.10:**
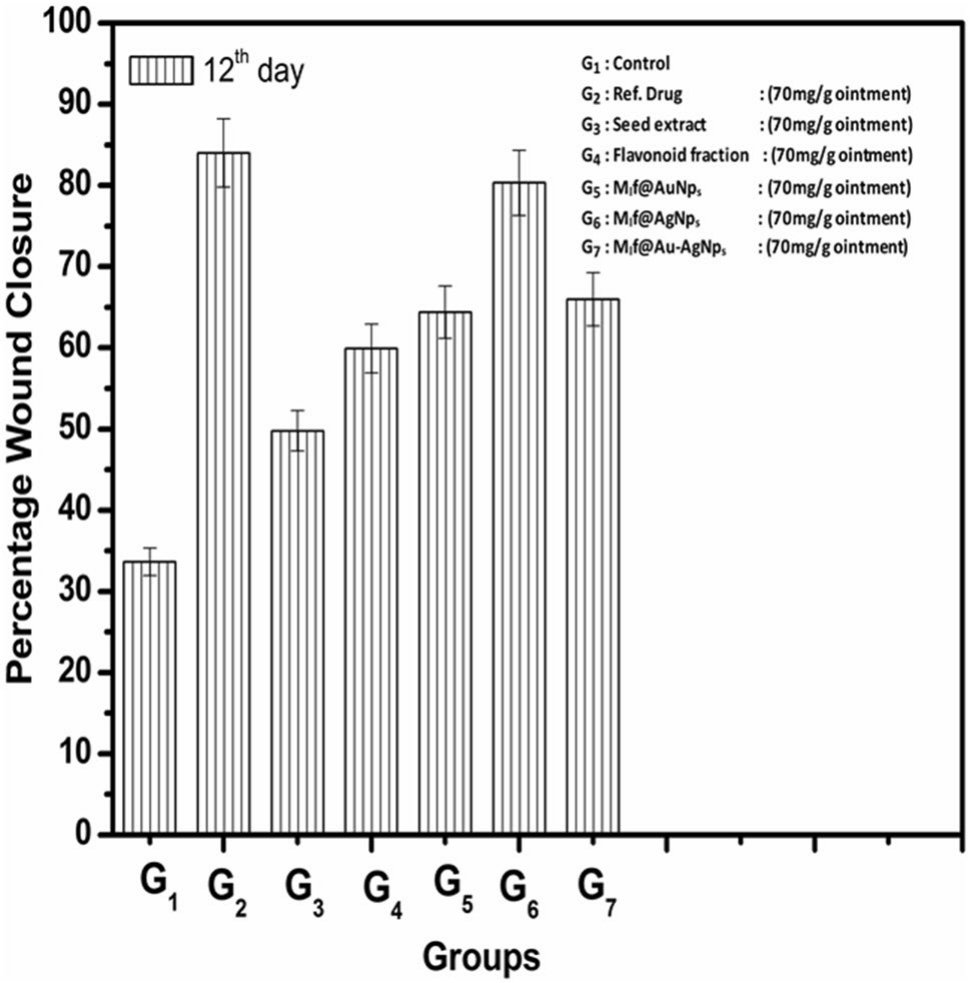
Wound closure % in various treatment groups

Percentage of wound closure in each case was calculated from the reduction in wound area (Fig. 10). The wound closure of seed extract (49.78%) was increased to a level of (59.93%) by flavonoids at the optimum dose (70 mg/g ointment). Interestingly, an increase in the percentage wound closure was induced by all the biofabricated mono and bimetallic nanoparticles at the same dose. The order of the percentage wound closure in different treatment groups was as follows: M_l_f@AgNp_s_ (80.33%) > M_l_@Au–AgNp_s_ (65.97%) > M_l_f@AuNp_s_ (64.37%), highlighting promising wound healing bio-efficacy of M_l_f@AgNp_s_. It seems the combination of silver and gold nanoparticles in the bimetallic M_l_@Au–AgNp_s_ is not enhancing wound healing bio-efficacy greater than M_l_f@AgNp_s._

## Discussion

Since ancient time, seeds of the plant *M. longifolia* are used as folk medicine for skin-associated ailments which indicate the presence of medicinally important secondary metabolites in the plant. Among the secondary metabolites, the polyphenolics and flavonoids have been reported responsible for dealing with skin-related issues (Ambiga et al. 2007; Lodhi and Singhai 2013; Pang et al. 2017). The detailed phytochemical analysis of this plant is lacking. The fact has encouraged us to isolate and characterize flavonoids from the seeds of the plant *M. longifolia*. LCMS-8030 studies of the column chromatographic fraction of native seed extract scanned in the lower (230–310) and higher (350–700) ranges of *m*/*z*, exhibited a family of seven flavonoids (Fig. 1a, b). The strong synergistic reduction potential of flavonoids present in the target plant (seeds) was used for the biofabrication of all the flavonoid-loaded nanoparticles and explored for wound healing bio-efficacy.

**Scheme 1:**
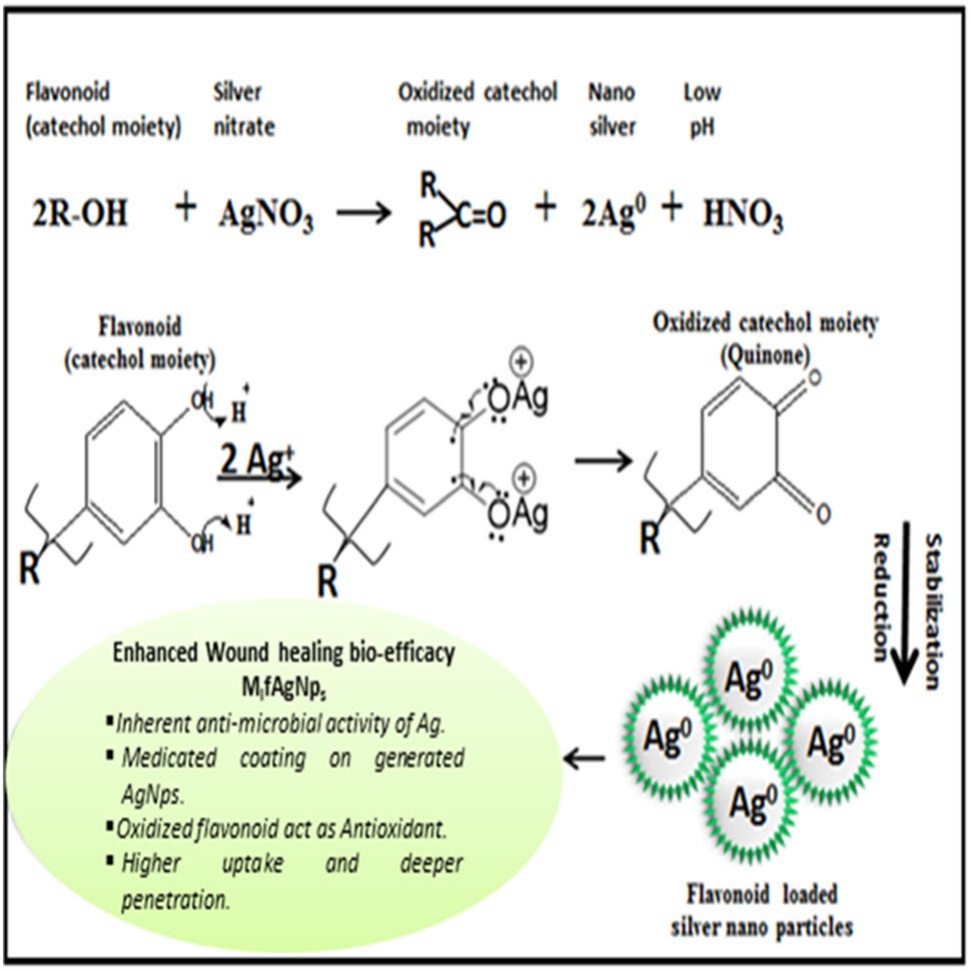
Proposed chemical reaction of flavonoid fraction with Ag^+^ ions rendering the formation of M_l_f@AgNp_s_

A tentative mechanism of reduction of Ag^+^ to Ag^0^ nano-state form is presented (Scheme 1). The bond dissociation energy (4.6–14.1 kcal/mol) of two –OH groups of catechol moiety of flavonoid is comparatively less (Trouillas et al. 2006) than normal phenolic –OH group (89.0 kcal/mol), facilitating the replacement of 2H^+^ with 2Ag^+^ ions and finally reducing into Ag^0^. Therefore, one catechol moiety of flavonoid molecule may reduce two silver ions (two protons per catechol) along with the corresponding quinone moiety. The oxidized quinone being electron deficient in nature may impart additional antioxidant bio-efficacy (free radical scavenging).

The wound healing is a complicated process involving competition in several skin components to permit the repair of damaged tissues. It is promoted by higher cellular uptake of antimicrobial agents enhancing deposition of collagen (increased level of hydroxyproline content), enzymatic interactions, regulation of matrix metalloproteinase enzyme, and pro-inflammatory factors (Shin et al. 2007; Prabhu and Poulose 2012; Caley et al. 2015). The cellular uptake of metal nanoparticles, in addition to the shape and size of nanoparticles, also depends (Zhang et al. 2008) on the extent of binding with the cell membrane (charge difference). Zeta potential values are often used as an indication of the stability of colloidal particles. The absolute values replicate the net electrical charge of the particles of functional groups present on the external surface (Aljabali et al. 2018). The negative value indicated the stability (repulsive barrier) of the nanoparticles preventing the agglomeration of nanoparticles (Patil et al. 2012). The negative potential, in the present case, therefore, might be arising from the loading of negatively charged functional groups (–OH groups of the flavonoids) (Somchaidee and Tedsree 2018). All the biofabricated nanoparticles possess negative charge (zeta potential) in the order: M_l_f@AgNp_s_ (− 22.5 mV) < M_l_f@Au–AgNp_s_ (− 31.5 mV) < M_l_f@AuNp_s_ (− 33.9 mV) indicating the least negative charge on M_l_f@AgNp_s_. The weak negative charge on silver nanoparticles is likely to encounter weak repulsion from strong electronegatively charged cell membrane and result into comparatively higher uptake. The uptake of M_l_f@AgNp_s_ ions is also facilitated by the strong tendency of Ag^+^ ions with thiol groups (–SH) of the cell membrane. Such enzymatic interactions are associated with the generation of ATP formation (Klueh et al. 2000). Silver catalyzes the reaction between the cellular O_2_ molecule and the H atom of (–SH) groups forming a disulfide bond (Ag–S–S–Ag) along with the formation of a water molecule instead of being consumed in structure, finally causing cell death (Davies and Etris 1997). Overall, the phenomenon allows accumulation and penetration of nanodrug into living tissues comparatively deeper. Further, a significant increase in the hydroxyproline content in healed tissues confirms the accumulation of collagen level and thus facilitates wound healing bio-efficacy. The enhanced percentage in wound closure (34.03%) compared to flavonoid (59.93%) also supports (Fig. 10) the promising wound healing efficacy of biofabricated M_l_f@AgNp_s_ (80.33%).

**Fig. 11:**
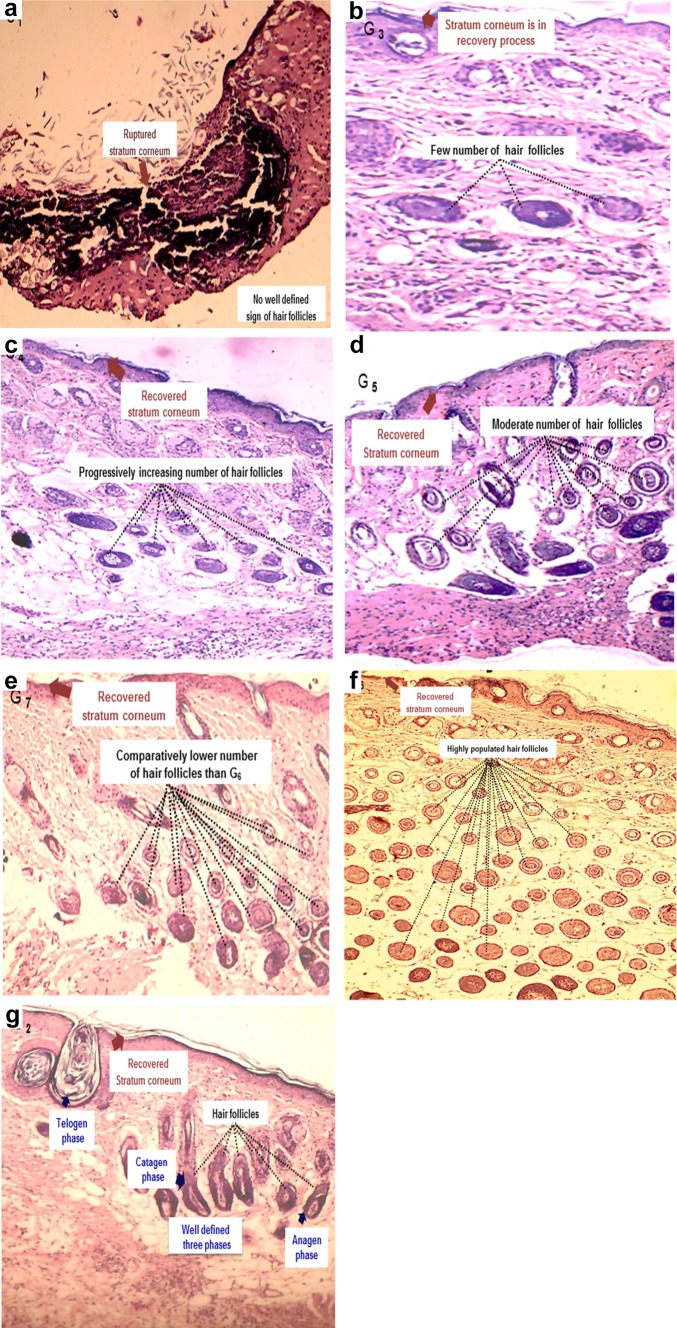
T.S. of skin: **a** control group, **b** seed extract, **c** flavonoid content, **d** M_l_f@AuNp_s_, **e** M_l_f@Au–AgNp_s_, **f** M_l_f@AgNp_s_, **g** reference drug at optimum dose 70 mg/g ointment, demonstrating progressive skin healing in terms of number of hair follicles and recovered stratum corneum

**Fig. 12:**
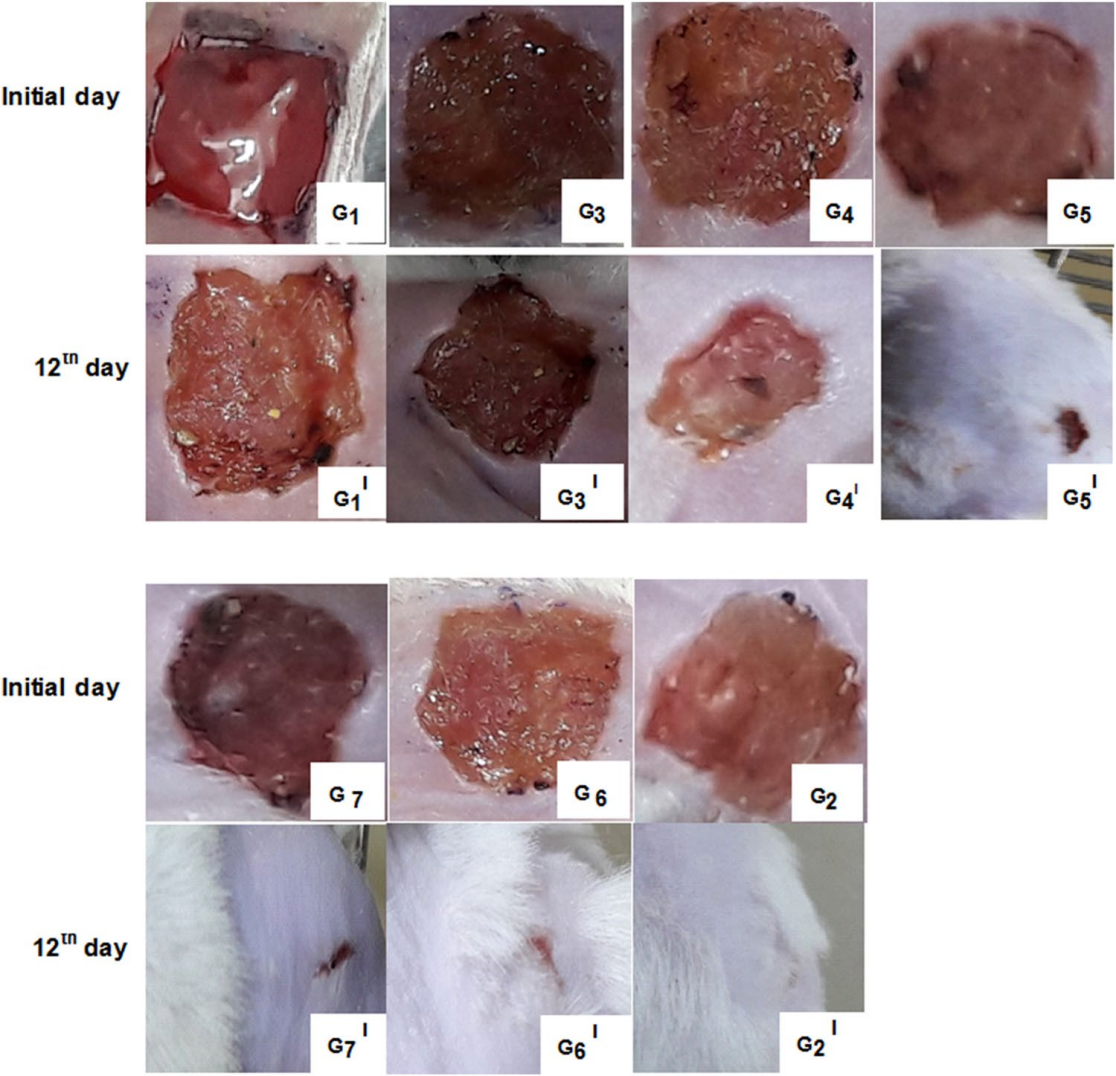
Photographs of skin on initial day and 12th day: (G_1_, G_1_^I^) control group, (G_3_, G_3_^I^) seed extract, (G_4_, G_4_^I^) flavonoid content, (G_5_, G_5_^I^) M_l_f@AuNp_s_, (G_7_, G_7_^I^) M_l_f@Au–AgNp_s_, (G_6_, G_6_^I^) M_l_f@AgNp_s_, (G_2_, G_2_^I^) reference drug at optimum dose 70mg/g ointment demonstrating progressive decrease in wound size

The skin healing tissues of all the treated groups were isolated on the (12th) day for histological evaluation. In the control group, no recovery appeared in ruptured stratum corneum. The M_l_f@AgNp_s_, exhibited trends of good recovery of stratum corneum with the progressively growing a number of well-defined hair follicles (Fig. 11a–c). However, reference drug-treated group revealed sound stratum corneum and fully developed hair follicles with all the three stages. Among the various treatments, M_l_f@AgNp_s_ showed faster wound closure rate (80.33%) and epithelization of the wound (18.00 days) (Table 1) with higher wound healing effects as compared to the native seed extract, flavonoid fraction, M_l_f@AuN_ps_, and M_l_ f@Au–AgNp_s_ (Fig. 12a–f).

**Table 1:** Effect of various treatments on wound healing parameters in the excision and incision wound models at an optimized dose

Groups optimized dose (70 mg/g ointment) except for control	Wound area (mm^2^) excision model	Epithelization period (days)	Breaking strength (g) incision model	Hydroxyproline (mg/g)
	12th day	–	10th day	15th day
1.Control	66.34 ± 1.70	33.00 ± 1.26	272.96 ± 1.30	30.21 ± 1.18
2. Placentrex	15.97*** ± 1.39	15.16*** ± 1.16	653.44*** ±1.05	80.23*** ± 1.44
3. Seed extract	50.63 ** ± 1.02	27.00*** ± 1.67	312.21 ** * ± 1.06	40.22*** ± 0.75
4. Flavonoid fraction	40.06*** ± 1.06	24.50*** ± 1.51	467.43*** ± 1.26	62.45*** ± 1.92
5.M_l_f@AuNp_s_	35.62*** ± 1.65	21.33*** ±1.50	612.85*** ±1.80	68.11*** ± 0.77
6.M_l_f@AgNp_s_	19.67*** ± 1.08	18.00*** ± 1.41	624.23*** ± 1.30	76.23***± 1.38
7. M_l_f@Au–AgNp_s_	34.02***±1.62	21.00*** ±1.50	616.01*** ± 1.55	70.03*** ± 1.42

Each value is the mean ± SD of six mice (*n* = 6) at ***p < 0.001

## Conclusions

A stable, simple and eco-friendly technique of biosynthesizing silver (M_l_f@AgNp_s_), gold (M_l_f@AuNp_s_) and bimetallic gold–silver (M_l_f@Au–AgNp_s_) nanoparticles loaded with flavonoids extracted from the seeds of the plant* M. longifolia* were effectively established under ambient conditions. The presence of a family of seven flavonoids in the seed extract of the plant was ascertained using LCMS-8030 analysis. The flavonoids played the major role in the reduction and capping during biofabrication of nanoparticles. All the biofabricated nano-biomaterials were thoroughly characterized. UV–Vis spectroscopy confirmed the surface plasmon resonance band of M_l_f@AgNp_s,_ M_l_f@AuNp_s,_ and M_l_f@Au–AgNp_s_ at *λ*_max_ = 432, 534 and 470 nm, respectively. XRD revealed that all biofabricated nanoparticles were of cubic symmetry. The FE-SEM images reported spherical shapes. The presence of silver, gold and both Au and Ag in the respective nano-biomaterials were confirmed by EDX spectra. TEM analysis reported the accurate size of (M_l_f@AgNp_s_: 35–50 nm; M_l_f@AuNp_s_: 36–60 nm and M_l_f@Au–AgNp_s_: 34–66nm). The surface charge (zeta potential) on the nanoparticles were found M_l_f@AgNp_s_ (− 22.5), M_l_f@AuNp_s_ (− 33.9), and M_l_f@Au–AgNp_s_ (− 31.9). In vivo healing parameters (wound area, wound closure, epithelization period, skin breaking strength and hydroxyproline content) have been examined in *Swiss albino* mice models. Among all biofabricated nanoparticles, M_l_f@AgNp_s_ showed significant enhancement (30.40%) in wound healing bio-efficacy compared to native seed extract. The enhanced wound healing potential of M_l_f@AgNp_s_ was assigned to the inherent antimicrobial property of Ag, zeta potential difference, the large surface area of nanoparticles, and coating of medicinally important flavonoid content on the nanoparticles. The oxidized flavonoids (quinone moiety) being electron deficient also impart additional antioxidant properties. The flavonoid-loaded silver nanoparticles have valuable future and open a novel channel for the development of effective complimentary herbal nanomedicine.

## Abbreviations

M_l_f@AuNp_s_: *Madhuca longifolia* flavonoid-loaded gold nanoparticles
M_l_f@AgNp_s_: *Madhuca longifolia* flavonoid-loaded silver nanoparticles
M_l_f@Au–Ag Np_s_: *Madhuca longifolia* flavonoid-loaded gold–silver bimetallic nanoparticles
